# Physically Sufficient Neural Mechanisms of Consciousness

**DOI:** 10.3389/fnsys.2019.00024

**Published:** 2019-07-04

**Authors:** Matthew Owen, Mihretu P. Guta

**Affiliations:** ^1^Department of Philosophy, Gonzaga University, Spokane, WA, United States; ^2^Department of Philosophy, Biola University, La Mirada, CA, United States; ^3^Department of Philosophy, Azusa Pacific University, Azusa, CA, United States

**Keywords:** neural correlate of consciousness (NCC), consciousness, theoretical approaches, sufficiency, unresponsive wakefulness syndrome (UWS), Alzheiemr’s disease, brain organoids

## Abstract

Neural correlates of consciousness (for brevity NCC) are foundational to the scientific study of consciousness. Chalmers (2000) has provided the most informative and influential definition of NCC, according to which neural correlates are minimally *sufficient* for consciousness. However, the sense of sufficiency needs further clarification since there are several relevant senses with different entailments. In section one of this article, we give an overview of the desiderata for a good definition of NCC and Chalmers’s definition. The second section analyses the merit of understanding the sufficiency of neural correlates for corresponding consciousness according to three relevant types of sufficiency: logical, metaphysical, and physical. In section three, a theoretical approach to consciousness studies is suggested in light of the sense in which NCC are sufficient for consciousness. Section four addresses a concern some might have about this approach. By the end, it will become apparent that our conception of NCC has important implications for research methodology, neuroethics, and the vitality of the search for NCC.

## Introduction

The search for neural correlates of consciousness (NCC) is integral to the science of consciousness (see Metzinger, 2000b; Kandel and Hudspeth, 2013, p. 18; Hohwy and Bayne, 2015; Frith and Rees, 2017, pp. 161–163; Overgaard, 2017; Storm et al., 2017). This search for the neuronal mechanisms at the mind-brain hinge has been marching on steadily for several decades (see Crick and Koch, 1990). Yet these are still relatively early days in an audacious endeavor, and thus further conceptual clarity is still in order.

*Sufficiency* is a key concept in an adequate definition of NCC (Koch, 2004, p. 97). However, there are multiple senses in which something might be “sufficient” since there are multiple types of sufficiency. The aim of this article is to clarify the sense in which NCC are sufficient for consciousness. For this has significant implications pertaining to research methodology, neuroethics, and the vitality of NCC research itself, which will become apparent throughout the article.

Chalmers (2000) has provided the most perspicuous definition of NCC, according to which neural correlates are sufficient for consciousness (see Koch, 2019, ch. 5). In the first section of this article, overviews of Chalmers’s definition and the desiderata for a good definition of NCC are provided. The second section analyses the merit of understanding the sufficiency of NCC according to three relevant types of sufficiency: logical sufficiency, metaphysical sufficiency, and physical sufficiency. This analysis demonstrates that the definition of NCC is best off if neural correlates are defined according to physical sufficiency.

However, the identification of NCC so defined will provide a limited amount of information that is insufficient by itself to fully address particular issues consciousness researchers are interested in. For this reason, in section three, a theoretical approach to consciousness studies is suggested. This approach allows for a theoretical framework to provide additional information upon which further inferences regarding consciousness can be based. To elucidate the practical application of such an approach, two examples are provided. The first concerns an unresponsive patient and what the Integrated Information Theory and the Global Neuronal Workspace (GNW) theory would predict about the patient’s state of consciousness given the neural activity present. The second example concerns Alzheimer’s disease and what the inability to consciously recall memories implies about the persistence of the self in light of two different theoretical frameworks, the psychological continuity view of the self and the Mind-Body Powers model of NCC. Section four addresses the concern that the proposed theoretical approach will philosophically pollute consciousness science.

## Defining NCC

To date, the most influential definition of NCC is found in Thomas Metzinger’s (2000b) edited volume *NCC: Empirical and Conceptual Questions*^1^. In “What Is a Neural Correlate of Consciousness?” Chalmers (2000) points out that there are various conceptions of NCC within the research literature. So he aims to provide conceptual clarity by giving a theoretically neutral, reasonable and coherent definition that reflects common usage (Chalmers, 2000, pp. 31, 38). This section clarifies the desiderata for the definition of NCC and then briefly explicates Chalmers’s definition. This will lay the foundation for analyzing the concept of “sufficiency” central to the definition in the following section “Analyzing Sufficiency”.

## Desiderata

The desiderata for a definition of NCC are the standards it needs to meet to be an adequate definition. Chalmers (2000, pp. 31, 38) briefly suggests several desiderata but without much elaboration. As mentioned above, he aimed for a definition that: (1) fits with common usage; (2) is reasonable and coherent; and (3) is theoretically neutral. We assume the same desiderata, but since adequately exploring the merit of different senses of sufficiency depends on these desiderata, a clarification of each is in order.

The first desideratum is that the definition comports with standard usage of the term within the NCC research community. When Chalmers proposed his definition for NCC there were fewer relevant publications. Since then there has been an explosion of literature and studies pertaining to NCC. As Koch (2019, ch. 5) points out: “Over the years, the deceptively simple concept of “neuronal correlates of consciousness” has been dissected, refined, extended, transmogrified and dismissed.” Consequently, it is no small task to verify that this desideratum of cohering with standard usage has been met. In an effort to do so reasonably, we must first consider the use and understanding of the term by leading researchers. One indicator that Chalmers’s definition meets this desideratum is Koch’s (2019, ch. 5) acknowledgment that his own preferred operational definition was “Helped along by Chalmers’s more rigorous formulation….” And although Sascha Benjamin Fink (2016, p. 3) finds Chalmers’s definition unsatisfactory and offers an alternative, he acknowledges its wide embrace:

*Most follow Chalmers in his outline: Mormann and Koch (2007) talk about “neural mechanisms or events which are sufficient for consciousness, ” and similar wording can be found in Hohwy and Frith (2004), Block (2005, p. 46), Hohwy (2007, 2009), Tononi and Koch (2008), Aru et al. (2012), Bayne and Hohwy (2013) and others. Although none of the mentioned authors quotes Chalmers (2000), the similarities are obvious. It is therefore reasonable to assume that the Chalmers-NCC is the default understanding of “NCC” in the neuroscience of consciousness^2^*.

The second desideratum is that the definition be reasonable and coherent. On the one hand, it should be internally coherent. If there is a logical contradiction within the definition itself, then it would be rendered useless. On the other hand, it would also be problematic if the definition is clearly contradictory with external knowledge. For example, suppose Hume’s (2007, p. 55) anti-realist^3^ view of causation was a proven fact but the definition of NCC fundamentally hinged on a realist view of causation. The implication would be that the referent “NCC” would not refer to anything that actually exists, which would sabotage NCC research. Thus, the definition must be internally and externally coherent.

The third desideratum is that the definition be theoretically neutral. Half a century before the contemporary search for NCC began, in an article entitled “The cerebral cortex in man: the cerebral cortex and consciousness,” Penfield (1938, p. 417) wrote:

*It seems quite proper that neurologists should push their investigation into the neurologic mechanism associated with consciousness and should inquire closely into the localization of that mechanism without apology and without undertaking responsibility for the theory of consciousness*.

Penfield is presenting a vision of brain science that studies the neuronal mechanism of consciousness but does not require researchers to commit to a theory of consciousness in order to identify the mechanism (see Penfield, 1975, pp. 4, 47 and 114). Given such neutrality, the legitimacy of such research does not hinge upon the truth or acceptance of a particular view of consciousness.

Today there is a myriad of views about the mind and consciousness embraced by influential philosophers and neuroscientists alike (see Haldane, 1998; Koons and Bealer, 2010; van Gulick, 2017). Especially in such a context, it is to the benefit of NCC research if the definition of NCC avoids tethering NCC research to a particular view of consciousness^4^. In this regard, Koch’s remarks hit home: “Note that the NCC themselves are neutral from the point of view of physicalism/materialism or one of the various shades of dualism. Under any reading, consciousness will have physical correlates.^5^” For that to be true, the definition of NCC must be theoretically neutral with respect to metaphysical views about the nature of consciousness.

The definition of NCC should also be theoretically neutral in another sense. It should not entail any particular empirical neurobiological conclusion about NCC, such as whether NCC is predominantly in the front or back of the brain (see Boly et al., 2017). It should leave such empirical issues open for empirical investigations to settle. This will not only assure that empirical matters are left to be decided by empirical research, but also prevent NCC research as a whole from being tethered to a particular empirical hypothesis. Having clarified the desiderata for a good definition of NCC (see Table 1), let us turn to Chalmers’s definition.

**Table 1:** NCC Definition Desiderata.

Reflects standard Use	Reasonable and coherent	Theoretically neutral
The definition comports with how the term is used within the NCC research community.	*Internal coherence*: The definition is internally coherent in that it avoids being internally contradictory.	*Metaphysical neutrality*: The definition does not entail a particular metaphysical view about the nature of consciousness.
	*External coherence*: The definition is coherent with information relevant to NCC research.	*Empirical neutrality*: The definition does not entail a particular empirical theory about the nature of specific NCC.

## Chalmers’s Definition

Before providing his own definition (Chalmers 2000, p. 17–18) first considers a basic definition of an NCC: “A neural system N is an NCC if the state of N correlates directly with states of consciousness.” This definition elicits three questions that guide Chalmers’s (2000, p. 18) definition:

1. What conscious states are relevant?
2. What neural states are relevant?
3. What does it mean for the neural states to correlate directly with conscious states?

Regarding question 1 Chalmers (2000, pp. 18–23) surveys multiple classes of phenomenal consciousness considered in the NCC literature^6^. The first is being conscious vs. not being conscious. The second is a background state of consciousness that is an overall state of consciousness at a particular time. The third is contents of consciousness such as perceiving Theresa May’s face on the television screen. The fourth is arbitrary phenomenal properties which can include specific states of any of the above classes and might be useful for a general definition of an NCC that applies to all classes of phenomenal consciousness.

Regarding question 2, the relevant neural states can depend on whether the neural correlate is the so-called “full NCC” or a “content-specific NCC” (Koch et al., 2016a, p. 308). Content-specific NCC are neural correlates of particular conscious states that include content. By contrast Koch et al. (2016a, p. 308) define the full NCC as: “The neural substrate supporting experience in general, irrespective of its specific content.” The full NCC can be the NCC of simply being conscious or the NCC of one’s overall conscious experience that may include multiple content specific conscious states.

Regarding question 3 Chalmers (2000, p. 24–28) asks two more fundamental questions about the nature of the direct correlation:

1. Must the neural state be necessary, sufficient, or necessary and sufficient for the conscious state it is correlated with?
2. Must the correlation hold across all cases or only across specific types of cases (i.e., cases with ordinary brain function in an ordinary environment, cases with a normal brain but unusual inputs, cases with varying stimulation, or cases with abnormal brain function due to lesions)?

As for question 3.1 Chalmers (2000, p. 32) reasons that an adequate definition of an NCC should not rule out the possibility of a conscious state having different neural correlates. Prior to empirical investigation, we do not know whether there is multiple realizability within a single human brain. Perhaps someone can have the conscious state of perceiving a face that corresponds with the activity of one coalition of neurons, N^1^, then lose that coalition, after which the individual’s perception of a face will correspond to the activity of another coalition of neurons, N^2^ (see Koch, 2004, p. 97 footnote 24). Whether this is possible is not the point^7^. Rather, the point is that whether or not it is possible should be decided by empirical neuroscientific investigation, as opposed to a pre-empirical, *a priori* definition. But if NCC are, by definition, *necessary* for the corresponding conscious state then such a scenario would be ruled out by definition. Consequently, the definition would lack theoretical neutrality. Hence Chalmers (2000, p. 32) does not define NCC as “necessary” but rather as “sufficient” (see also Koch, 2004, p. 97 footnote 24).

As for question 3.2 Chalmers (2000, pp. 31) thinks that the conditions most relevant to NCC include normal brain function that can allow for some atypical inputs and brain stimulation as long as there are no changes to brain structure. He points out that lesion studies are repeatedly used to make inferences about neural correlates but reasons that such methodology is flawed since “the identity of an NCC is arguably always relative to specific brain architecture and normal brain functioning, and correlation across abnormal cases should not generally be expected” (2000, pp. 32). Others might agree that caution is necessary, but think that lesion studies can nevertheless provide important information especially when the findings from a lesion study are coupled with and corroborated by other studies, such as stimulation studies in a healthy brain (see Koch et al., 2016a, p. 308)^8^.

Having considered the above questions, and given the aforementioned desiderata Chalmers (2000, p. 31) defines a neural correlate of consciousness as follows^9^.

*An NCC is a minimal neural system N such that there is a mapping from states of N to states of consciousness, where a given state of N is sufficient, under conditions C, for the corresponding state of consciousness*.

An NCC is a minimal neural system that is sufficient under certain conditions for the corresponding state of consciousness (see Koch et al., 2016a, p. 307). While Chalmers does not include the notion of necessity, he does include sufficiency, which is analyzed in section “Analyzing Sufficiency” Yet, before focusing on that particular facet of the definition it will be helpful to briefly clarify other aspects^10^.

The phrase “*minimal* neural system” prevents neural processes that enable the specific neural correlate from being included as part of the NCC (Chalmers, 2000, p. 24). With regards to NCC, we are not concerned with everything taking place in the nervous system when one is in a particular conscious state but rather the minimal neural states that correspond to a particular conscious state. The qualifier “under conditions C” allows for the fact that there are enabling conditions that allow NCC to function properly. The phrase “there is a mapping from states of N to states of consciousness” pertains to subjects across a species, not just an individual member. However, this mapping across a species is not necessarily a correspondence of identical neural states in every subject. The search for NCC is a search for biological regularities, not necessarily identical correspondence relations (for no two brains, not even of twins or even clones, are exactly alike)^11^. Biological regularities of all kinds permit some variation. Furthermore, there are phenomenological variations to consider. If shown an image of the Eiffel Tower, two men could report seeing the image, but the one who met his wife at the tower will likely have a different overall conscious experience as he perceives the image than the one who has never been to Paris. Given such variations, we should not expect the search for NCC to reveal correlations that have zero variation across a species, but rather neurobiological correlations reflecting biological regularities^12^.

To summarize: an NCC, as defined by Chalmers (2000, p. 31), is a minimal neural system that is *sufficient* under certain conditions for the corresponding state of consciousness. Next, the sense in which NCC are sufficient will be considered.

## Analyzing Sufficiency

Striking a good balance between clarity and complexity, Chalmers’s definition has served NCC research well for nearly two decades. Yet there remains a need for further clarification regarding the sense in which neural correlates are *sufficient* for consciousness. For just as there are various senses of necessity corresponding to different types of necessity, there are various senses of sufficiency corresponding to different types of sufficiency.

In this section, the merit of defining NCC according to three relevant senses of sufficiency is considered in light of the desiderata for a definition of NCC. First logical sufficiency is considered, then metaphysical sufficiency, and finally physically sufficiency. For the purpose of this article we analyze the definition of NCC in light of all three while acknowledging that philosophers disagree on whether metaphysical sufficiency is a distinct type of sufficiency. This issue, however, does not affect our final conclusion.

### Logical Sufficiency

Logical sufficiency is fundamentally due to logical entailments, as opposed to the nature of entities or natural regularities. In order to further grasp the concept of logical sufficiency, let us first consider logical necessity, which logical sufficiency mirrors. *X* is *logically necessary* for *Y* if, and only if, *Y* is *logically impossible* without *X*. The claim that Hilbert is a married bachelor is not logically impossible because Hilbert cannot find a date, but rather because a bachelor is by definition unmarried. Therefore, it is logically necessary that Hilbert be unmarried given that he is a bachelor. And it is logically impossible for Hilbert to be married given he is a bachelor. After all, given the definition of a bachelor, the propositions “Hilbert is a bachelor” and “Hilbert is married” entail Hilbert is not married and married, which is a contradiction (assuming the propositions refer to the same person at the same time).

Relying on the concepts of logical necessity and logical impossibility, let us describe logical sufficiency in the present context as follows:

**X* is *logically sufficient* for *Y* if, and only if, given *X* then *Y* is logically necessary and not-*Y* is logically impossible*.

Once again, the concept can be elucidated *via* examples. The fact that Hilbert is a bachelor is logically sufficient for the fact that Hilbert is unmarried since a bachelor is by definition unmarried. And given that red is by definition a color, the bench being red is logically sufficient for the bench being colored.

Now we can consider what it would mean for a neural correlate of consciousness to be logically sufficient. Neural state N^1^ is logically sufficient for the conscious state C^1^ if, and only if, given N^1^ then C^1^ is logically necessary and C^1^ not manifesting is logically impossible. While the common example of firing C-fibers as the neural correlate of pain is empirically inaccurate, we can use it to provide elucidation in the present context. Suppose the definition of “firing C-fibers” logically entailed the conscious state of pain. Given that and the presence of firing C-fibers, it would follow out of logical necessity that pain is present. There would be no noncontradictory way around it—if C-fibers are firing, pain is present—assuming that the definition of firing C-fibers entails pain so firing C-fibers are logically sufficient for pain. If NCC were logically sufficient for corresponding consciousness, there would be profound implications.

To begin with, it would significantly affect the methodology in the search for NCC. If NCC were logically sufficient, identifying the conscious state corresponding to a neural correlate could be a matter for *a priori* philosophical investigation devoid of any *a posteriori* empirical investigation. For clarification, “a priori knowledge” can be known by reason alone apart from experience. One can know *a priori* that 2 + 2 = 4, or that Q is true given that “if P is true then Q is true” and “P is true.” By contrast, “a posteriori knowledge” is gained through our sense experience of the world around us.

Consciousness research includes both types of reasoning (Guta, 2019); and some things can be known by *a priori* reasoning alone, whereas other things require empirical investigation. For an example of the former, if we know that a subject has a content specific conscious state, such as seeing a face, then the subject must have an overall background state of being conscious. No empirical investigation is needed to know this; it follows as a logical entailment that the subject is conscious from the fact that the subject is conscious of a face. A content-specific conscious state is logically sufficient for being conscious, so we can simply deductively reason to the conclusion that the subject is conscious. By contrast, consider how researchers identify the conscious state that corresponds to a particular NCC. They must know what state the neurons of the brain are in and what conscious state a subject is in. Using functional brain imaging researchers can observe neuronal activity in a subject’s brain, and through reports from a subject, researchers can know a subject’s conscious state. To know what neural state correlates with the conscious state of perceiving a face, researchers try to identify what particular neural state(s) is present when the subject perceives the face. The process of identifying the correspondence relation between the conscious state and its neuronal footprint depends on this empirically gathered information, gained from brain imaging and listening to reports^13^.

However, if NCC were logically sufficient, the methodology could be very different. In principle, researchers could logically deduce what conscious state was present simply by knowing what neural state is present. The corresponding conscious state could be known by deductive reasoning *via* logical entailments if the neural state were logically sufficient. There would be no need for verbal reports to discern what conscious state the subject is in, or any other empirically identifiable physiological indicator (see Tsuchiya et al., 2015). In other words, what conscious state corresponds could be known *a priori* by following logical entailments and rules of logic, given knowledge of what neural state is present. The correspondence would not need to be empirically discerned but could be known *a priori* by deductive reasoning alone.

But such *a priori* methodology, which would depend on NCC being logically sufficient, does not comport with the a posteriori empirical methodology actually used by researchers to identify the correspondence between neuronal and conscious states. The methodology used presupposes that the correspondence cannot be logically deduced, but is rather known through an empirical process. At present, the process involves using brain imaging to identifying neural states present when conscious states are present, which is known from verbal reports or physiological indicators. There is room in this process for inductive, abductive, and deductive reasoning. But it is not a process of purely deductive reasoning along logical entailments from knowledge of the neural states present, which it could be if NCC were logically sufficient for corresponding consciousness. Thus, defining NCC as logically sufficient does not comport with the concept of NCC informing the methodology NCC researchers actually use.

A second problem pertains to the second desideratum. If “sufficient” means “logically sufficient” the definition would not be internally incoherent, but it would suffer from being externally inconsistent with the logical possibility of zombies. Chalmers (1996, p. 94) describes a zombie as something physically the same as a conscious being such as himself, but with no conscious experience. Christof Koch and Crick (2001) applied a rendition of this concept to what they call “zombie agents”; that is, sensorimotor neural systems in the primate brain that can be integral to actions but nevertheless unconscious; and as they clarify: “By “unconscious” we mean any neuronal activity that does not give rise to conscious sensation, thought or memory” (Koch and Crick, 2001, p. 893)^14^. The existence of zombie agents is important to the search for NCC because it raises a key question: what is the difference that makes the difference between neuronal activity that is sufficient for consciousness and that which corresponds to the unconscious (Crick and Koch, 2003, p. 120; Koch, 2012, p. 90)?

What matters for the purported logical sufficiency of NCC is not whether zombies are real or physically possible nor metaphysically possible, but whether they are merely logically possible. They are logically possible if the concept of a zombie is logically consistent. Philosophers often articulate modal claims regarding possibility and necessity in terms of “possible worlds” semantics. Accordingly, zombies are logically possible if a possible world with zombies is logically coherent, devoid of logical contradiction. While such a world might be very different than the actual world and have different laws of nature, it does not seem logically inconsistent. Hence, it is often thought by philosophers that zombies are at least logically possible, regardless of whether they are physically possible in the actual world. Here’s the implication for a definition of NCC: if neural correlates are logically sufficient for the corresponding consciousness, then zombies are logically incoherent, but it does not seem that they are logically incoherent. Therefore if “sufficient” in the definition of NCC means “logically sufficient,” the definition is at risk of failing to meet the desiderata of being both internally and externally coherent. The definition would create an unfortunate conflict: if one thought zombies are logically possible, they would be inclined to think the concept of NCC is not coherent and *vice versa*.

Since talk of zombies might sound trivial, let us consider another example related to neuroethics. In 2011, at the Institute of Molecular Biotechnology in Vienna a postdoctoral researcher, Madeline Lancaster, inadvertently brought about the production of a brain organoid from human embryonic stem cells (Willyard, 2015, p. 520). The brain organoids neuroscientists can now grow consist of several million neurons. In April 2018, *Nature* published an article on the ethics of experimenting with brain organoids. The team of authors, led by Farahany et al. (2018, p. 430), offered the following description of brain organoids:

*Brain organoids can be produced much as other 3D multicellular structures resembling eye, gut, liver, kidney and other human tissues have been built. By adding appropriate signaling factors, aggregates of pluripotent stem cells (which have the ability to develop into any cell type) can differentiate and self-organize into structures that resemble certain regions of the human brain*.

These so-called “mini-brains” resemble human brains in noteworthy ways regarding their constitution, neural activity, and structure. Thus, they prompt a key question with serious ethical implications: are they conscious? The question is natural to ask because it seems possible that despite them being composed of human brain tissue, and having similar structural features, and neural activity, it is possible they are not conscious. To use Chalmers’s terminology, it’s possible they are “zombie” mini-brains.

Brain organoids are much smaller and far less developed than actual brains, but imagine a scenario in which a full human nervous system is grown. It would still seem reasonable, if not all the more pertinent, to ask: is it conscious? And the question is sensible because it seems at least logically possible that such a nervous system would not be sufficient for consciousness. Yet, it would seem that such would not even be logically possible if NCC are logically sufficient for consciousness. One could appeal to the clause in Chalmers’s definition “under conditions C” and claim that the neural correlates in such a nervous system would be logically sufficient for consciousness under the correct conditions. This is a fair point, but it actually highlights the relevance of zombies. A zombie would meet all the appropriate physical conditions and yet it still seems logically possible for consciousness to be lacking despite the presence of sufficient NCC under the correct physical conditions. And this logical possibility suggests that claiming NCC are logically sufficient for corresponding consciousness, given suitable physical conditions, is misguided. Thus, the mere logical possibility of zombies makes it difficult for the definition of NCC understood according to logical sufficiency to meet the second desiderata of cohering with relevant external information—i.e., zombies are logically possible.

Before concluding this section, it is also worth noting how the definition would fail to be theoretically neutral. As eluded to above Chalmers (2000, p. 32) does not define NCC as *necessary* for corresponding consciousness in order to leave open the possibility of a conscious state having different neural correlates (see Koch, 2004, p. 97 footnote 24). This is a matter to be decided by empirical investigation, not by definition and *a priori* reasoning based on the meaning of the definition. The same is true for whether or not an NCC could be the correlate of multiple conscious states. The former issue pertains to the possibility that a conscious state could correlate with multiple neural states. For example, could pain possibly correlate with C-fibers firing and another neural process involving different neurons rather than C-fibers? This issue we are focusing on now is the reverse, the possibility that a neural state could correlate with multiple conscious states. For example, could C-fibers firing correlate with pain and another conscious state rather than pain? This possibility could be conceptually ruled out with no need for empirical investigation if NCC were by definition logically sufficient for the corresponding conscious state. For if C-fibers firing were the NCC of pain and therefore logically sufficient for pain, then C-fibers firing would necessarily entail the correlated conscious state—pain. As a result, it would not be possible for C-fibers firing to be correlated with some other conscious state instead of pain. It could be conceptually ruled out, but it seems this is an issue that should be empirically settled. However, if NCC are defined as logically sufficient for corresponding consciousness then such issues could be settled pre-empirically, so the definition would lack empirical neutrality.

In sum, if “sufficient” in the definition of NCC means logically sufficient the definition would have problems with each desiderata (see Table 2). With respect to the first desiderata of reflecting standard usage, it would be inconsistent with the concept of NCC that informs the empirical methodology NCC researchers use. Regarding the second desiderata of being reasonable and coherent, it would lack external coherence insofar as the concept of NCC would be inconsistent with the logical possibility of zombies. And as for theoretical neutrality, the definition would logically negate the possibility that a neural correlate might correlate with multiple conscious states which should be left to empirical investigation to confirm or deny. Thankfully, for Chalmers’s definition, there are two other relevant senses of “sufficient.”

**Table 2:** NCC defined according to logical sufficiency.

**Logically sufficient NCC**
Neural state N^1^ is logically sufficient for conscious state C^1^ if, and only if, given N^1^ then C^1^ is logically necessary and ¬C^1^ is logically impossible.		
**Reflects standard use**	**Reasonable and coherent**	**Theoretically neutral**
Lacks consistency with the concept of NCC that informs the empirical methodology NCC researchers employ.	Lacks external coherence as it is inconsistent with the logical possibility of zombies.	Lacks empirical neutrality since it logically rules out prior to empirical investigation the possibility of a neural state correlating with two different conscious states.

### Metaphysical Sufficiency

The second relevant sense of sufficiency worth considering is metaphysical sufficiency, which pertains to the natures of things. Once again, it will help to first clarify metaphysical necessity, which metaphysical sufficiency mirrors. *X* is *metaphysically necessary* for *Y* if, and only if, the nature of *X* requires *Y*.

Regarding freewill, someone might claim a free action requires the ability to do otherwise since it is essential to the nature of a free action. On this view, the ability to do otherwise could be described as metaphysically necessary for freewill. In philosophy of mind, some say mental causation is impossible for a nonphysical mind due to the nature of causation, which requires spatial extension (for example see Kim, 2005, ch. 3). Spatial extension is allegedly metaphysically necessary for causation and thus mental causation is *metaphysically impossible* for nonphysical minds that are not spatial. Of course, there are substantive replies (see Bailey et al., 2011; Owen, 2018b). Nevertheless, these examples are helpful for introducing the concepts of metaphysical necessity and impossibility, which can be used to describe metaphysical sufficiency in the present context:

**X* is metaphysically sufficient for *Y* if, and only if, given *X* then *Y* is metaphysically necessary and not-*Y* is metaphysically impossible due to the natures of *X* and *Y**.

Metaphysical sufficiency is relevant to contemporary philosophical discussions about powers. For example, Nancy Cartwright and Pemberton (2013) argue that Aristotelian powers that produce specific effects due to their natures are central to modern science. Accordingly, a chemist discerns that H_2_O has the power to dissolve NaCl whenever the conditions are adequate (e.g., salt is placed in a cup of water). H_2_O causes NaCl to dissolve with regularity since the manifestation of this power is metaphysically sufficient for the effect. Given the nature of the power, when it is manifested in the appropriate conditions an effect of a particular nature follows.

Let us now apply metaphysical sufficiency to NCC. Neural state N^1^ is metaphysically sufficient for conscious state C^1^ if, and only if, given N^1^ then C^1^ is metaphysically necessary and C^1^ not manifesting is metaphysically impossible due to the natures of N^1^ and C^1^. If Chalmers’s definition of NCC is understood according to metaphysical sufficiency it would be better off than if it were understood according to logical sufficiency. For one, it would seem to comport more with what NCC researchers have in mind when they are looking for neural correlates. After all, researchers are concerned with what natural characteristics of particular neurons correspond to consciousness, which might pertain to something specific about their nature such as their morphological structure, molecular compositions, biophysical properties, or function (i.e., spiking rates, oscillation rhythms, spike synchronization, etc.). The definition would also be internally consistent.

Despite its strengths, however, the definition would still suffer from shortcomings that could be avoided. The zombies discussed in the previous section also raise trouble for NCC defined in terms of metaphysical sufficiency. To begin with, if zombies are not merely logically possible but also metaphysically possible, the definition of NCC would be inconsistent with such a possibility and therefore lack external consistency. For if a neural state N^1^ were metaphysically sufficient for conscious state C^1^ then it would be metaphysically impossible for N^1^ to be present without C^1^. But such would be metaphysically possible if zombies were metaphysically possible. To the definitions benefit, the metaphysical possibility of zombies is more debatable than the logical possibility of zombies and more difficult to demonstrate. Nevertheless, the definition of NCC would remain vulnerably dependent on the outcome of such debates if it is inconsistent with one side in the debate.

The definition would also lack theoretical neutrality in two ways. First, it would lack metaphysical neutrality because it would be inconsistent with versions of dualism that permit the metaphysical possibility of zombies. While this concern also pertains to the metaphysical possibility of zombies, it is not the same concern as that just mentioned above regarding external inconsistency. For that issue depends on the metaphysical possibility being a real and demonstrated possibility. This concern regarding metaphysical neutrality does not depend on the possibility being real or demonstrated, but rather on the fact that the possibility would be ruled out in virtue of how NCC are defined; and such an issue should not be decided by how NCC are defined. Yet more importantly for present purposes, this would entail a significant burden—the need to demonstrate that zombies are not metaphysically possible—in order to justify the metaphysical possibility of NCC and thus legitimate the search for NCC.

The second way the definition would lack theoretical neutrality pertains to empirical neutrality. In the previous section, we argued that the definition of NCC understood according to logical sufficiency would rule out the possibility that a neural correlate could possibly correlate with a different conscious state, which is an empirical matter that should be decided by empirical investigation rather than by definition. We gave the example of C-fibers firing correlating with pain and another conscious state rather than pain. The charge was that the definition would lack empirical neutrality since it would rule out this possibility prior to empirical investigation. The same would be true if the definition were understood according to metaphysical sufficiency. For if a neural state N^1^ were metaphysically sufficient for conscious state C^1^ then C^1^ would necessarily follow given the presence of N^1^ and it would be impossible for another conscious state C^2^ to follow instead of C^1^. This would be the case in every metaphysically possible world, including the actual world. Yet, again, whether N^1^ could be the NCC of either C^1^ or C^2^ should be decided by empirical investigation, not by definition.

All things considered, if “sufficient” in Chalmers’s definition meant “metaphysically sufficient” then the definition would be better off than if it meant “logically sufficient” (see Table 3). This is because the definition would comport well with standard usage and enjoy internal consistency. However, the definition would be inconsistent with the metaphysical possibility of zombies and it would lack empirical neutrality, two shortcomings that are best to avoid. Yet, there is a third sense of sufficiency worth considering.

**Table 3:** NCC defined according to metaphysical sufficiency.

**Metaphysically sufficient NCC**
Neural state N^1^ is metaphysically sufficient for conscious state C^1^ if, and only if, given N^1^ then C^1^ is metaphysically necessary and ¬C^1^ is metaphysically impossible due to the natures of N^1^ and C^1^.		
**Reflects standard use**	**Reasonable and coherent**	**Theoretically neutral**
Consistent with standard usage since researchers try to identify what characteristics of neurons are sufficient for consciousness, which might pertain to their *whole nature* or *something* *specific about their nature* such as their morphological structure, molecular compositions, biophysical properties, or function.	Lacks external coherence as it is inconsistent with the metaphysical possibility of zombies.	Lacks metaphysical neutrality since it rules out metaphysical views of consciousness that entail the metaphysical possibility of zombies. Lacks empirical neutrality, since it rules out prior to empirical investigation the possibility of a neural state correlating with two different conscious states.

### Physical Sufficiency

The third relevant sense of sufficiency is physical sufficiency. The natural and physical sciences focus on physical necessities and sufficiencies. In some respects, physical sufficiency is easier to grasp than the previous types of sufficiency since we are very familiar with it. Nevertheless, some precision is needed to clarify exactly what is meant by *physical sufficiency*.

In a reductivist milieu, “physical” is often taken as a referent to something regarding fundamental physics. But we do not here assume a reductivist view of physical objects. Subatomic particles are physical objects as are atoms, we assume, and likewise for trees, mammals, bodily organs, and cells. And physical laws pertaining to micro objects like the Higgs boson but also to macro objects like cells, organs, mammals, and trees. Laws of physics as well as biological laws are included^15^. Some might say we have neglected a relevant type of sufficiency that must be considered, namely nomological sufficiency, which focuses on natural laws. However, nomological sufficiency is not specifically addressed here since our view of physical sufficiency encompasses nomological sufficiency.

In describing physical sufficiency, once again, it will be helpful to begin with physical necessity, which physical sufficiency mirrors. *X* is *physically necessary* for *Y* if, and only if, *X* is required for *Y*, which is *physically impossible* without X, due to physical laws. For example, oxygen is necessary for human life, which is physically impossible without oxygen, due to physical laws. As for physical sufficiency, we can describe it in this context as follows:

**X* is physically sufficient for *Y* if, and only if, *X* satisfies the requirements of physical laws for *Y**.

If one physical event is physically sufficient for a second event, the second event follows with consistent regularity due to physical laws. A temperature of zero degrees Celsius is sufficient for H_2_O to change from a liquid state to a solid state. The structure of a Boeing 747 together with forces exerted by its fully functioning CFM-56 jet engines are sufficient for the airliner to fly through the atmosphere. Understanding specific physical conditions that are physically sufficient to bring about a particular kind of effect is vital to technological development, in which we intend to bring about specific kinds of effects with consistent regularity. It is also vitally important to the development of medical treatments where it is desired to eliminate particular diseases by eliminating the physical conditions physically sufficient for the disease.

Now let’s apply physical sufficiency to NCC. Neural state N^1^ is a neural correlate physically sufficient for conscious state C^1^ if, and only if, N^1^ satisfies the requirements of the physical laws in the actual world for C^1^. One significant benefit of understanding the sufficiency of neural correlates in terms of physical sufficiency pertains to the first desiderata. It clearly comports with how NCC researchers use the term. Correspondingly, it also fits with how the research in the search for NCC is carried out in an empirical manner as opposed to simply relying on logical deductions. For the search for NCC is aimed at identifying the physical states and processes at the level of neurons and coalitions of neurons that support consciousness. And these must be identified *via* rigorous empirical investigation in which certain neural candidates are postulated as NCC and empirically tested by mapping the conscious states of subjects with neural states identified *via* brain imaging. Sometimes these empirical postulates are verified and sometimes they are falsified.

What matters in the search for NCC are physical facts about the neural states sufficient for consciousness that are known *via* empirical investigation; they are not logically deducible *via a priori* reasoning alone. Given this, it is not surprising that Koch (2004, p. 205), who co-instigated the contemporary search for NCC with Crick^16^, would write:

*It does not feel like anything to be a zombie…Some argue that the logical possibility of their existence implies that consciousness does not follow from the natural laws of the universe, that it is an epiphenomenon. From this point of view, whether or not people feel makes no difference to themselves, to their offspring, and to the world at large. To Francis and me, this point of view seems sterile. We are interested in the real world, not in a logically possible never-never land where zombies roam. And, in the real world, evolution gave rise to organisms with subjective feelings*.

If the search for NCC is a search for the neural states physically sufficient for consciousness, then it is not threatened by the logical or metaphysical possibility of zombies.

A world with zombies might be logically and metaphysically possible. But what NCC research is focused on is what neuronal characteristics are physically sufficient for consciousness given the physical laws present in the actual world, not all logically or metaphysically possible worlds. This search is compatible with both the logical and metaphysical possibility of zombies since it is focused on what is physically sufficient given the physical laws of nature. It is aimed at uncovering the physical neuronal states corresponding to consciousness with consistent regularity, the regularity of biological laws. Some neural states correspond with the absence of consciousness, which Koch and Crick (2001) call “zombie agents,” while others regularly correspond with the presence of consciousness due to the physical laws of nature. The aim of NCC research is to distinguish the latter from the former (see Crick and Koch, 2003, p. 120; Koch, 2012, p. 90). In sum, when the definition of NCC is understood according to physical sufficiency the logical and metaphysical possibility of zombies does nothing to threaten it, and the idea of “zombie agents” can be a useful conceptual tool.

The definition also enjoys theoretical neutrality regarding an empirical issue that it would lack if understood according to the previous two senses of sufficiency. It would not decide prior to empirical investigation whether a particular neural state N^1^ could systematically correlate with one conscious state C^1^, or rather, a different conscious state C^2^. This issue is left open for empirical investigation to decide, as it discerns what the physical laws of nature permit. In addition, the definition enjoys theoretical neutrality metaphysically speaking in that the existence of physically sufficient NCC is compatible with a range of views in the metaphysics of mind—from physicalist views like the identity theory, to hylomorphism, to Cartesian dualism (see Block and Stalnaker, 1999; Swinburne, 2013; Owen, 2018a)^17^. Given this, the legitimacy of NCC research would be less affected by changing tides in philosophy of mind.

However, a word of caution is in order. It is easy to confuse physical sufficiency with a causal notion of neural correlation. Although a correlation is not necessarily indicative of causation. And if “sufficient” in the definition of NCC meant that every conscious state has a neural correlate that causes it to exist, then the definition would lack theoretical neutrality. At worst, it would entail epiphenomenalism, assuming the effect cannot be identical to its cause and that the neural state is always the cause of the conscious state. At best, but concerning nevertheless, the definition would prematurely rule out views that explain the correlations in terms of constitution, supervenience, grounding, or some other relation that might not be causal. So physical sufficiency should not be confused with physical causation, which is only one possible explanation of a physical sufficiency.

In the final analysis, if “sufficient” in Chalmers’s definition of an NCC is understood in terms of physical sufficiency, it would appear to meet each desiderata (see Table 4). It would fit common usage, be reasonable and coherent internally as well as externally, and be theoretically neutral both metaphysically and empirically.

**Table 4:** NCC defined according to physical sufficiency.

**Physically sufficient NCC**		
Neural state N^1^ is a neural correlate physically sufficient for conscious state C^1^ if, and only if, N^1^ satisfies the requirements of the physical laws in the actual world for C^1^.		
**Reflects standard use**	**Reasonable and coherent**	**Theoretically neutral**
Consistent with standard usage since researchers try to identify what characteristics of neurons *in the actual world given its physical laws* are sufficient for consciousness, which might pertain to their whole nature or something specific about their nature such as their morphological structure, molecular composition, biophysical properties, or function (i.e., spiking rates, oscillation rhythms, spike synchronization, etc.).	In addition to being internally consistent, the definition is externally consistent with the logical and metaphysical possibility of zombies. And “zombie agents” can conceptually aid the search for NCC interested in the physical differences that matter to consciousness.	Theoretically neutral as it is compatible with a range of views regarding the metaphysics of consciousness. Empirically neutral as it leaves open for empirical investigation the possibility of a neural state correlating with multiple, different conscious states.

## Theoretical Implications

The definition of NCC understood according to physical sufficiency meets all the desiderata for a good definition of NCC outlined in section Defining NCC. That said, NCC so defined might fail to meet all the expectations some consciousness researchers have for NCC.

A clear implication of NCC being physically sufficient is that, despite the pragmatic mental health benefits locating NCC can produce, the search for NCC will leave profound questions about consciousness unanswered. By themselves, NCC will not solve the hard problem of consciousness or tell us what its nature is (see Chalmers, 1995). Moreover, identifying neural correlates in humans we already know are conscious will not necessarily tell us what other candidates for being conscious are actually conscious. When NCC are properly understood it becomes apparent that the search for NCC is limited in its scope of what it can tell us about consciousness (see Chalmers, 1998; Koch, 2019, ch. 7; Owen, 2019b).

Many consciousness researchers are principally interested in the pragmatic benefits of identifying NCC. They want to identify neural states sufficient for undesired mental states such as anxious feelings, so we can treat mental illnesses by influencing their neural underpinnings. Such researchers can find the theoretical neutrality of NCC beneficial since it precludes NCC research from being tethered to issues like the hard problem and thus influenced by relevant debates. This can allow the search for NCC to march forward without being unduly hindered by slower progression in other areas of consciousness research.

That said, many consciousness researchers have additional aspirations, such as solving the hard problem and identifying empirical indicators of the presence of consciousness in unresponsive medical patients, animals, and perhaps machines. It is important to acknowledge the limitations of what NCC can tell us concerning such issues, to elucidate their epistemic role. Identifying the neural correlate of a human person’s feeling of pain will not tell us what exactly pain is, why such feelings exist, whether unresponsive medical patients feel pain, nor whether an iPhone feels pain. However, that does not entail NCC are irrelevant to these issues. NCC data is not sufficient by itself to answer certain questions, but when coupled with a warranted theoretical framework, NCC data provides important information for further inferences or extrapolations.

In this section, we illustrate the applicability of a theoretically motivated approach to consciousness research by considering two particular issues. To begin with, we consider the utility of applying a theoretical framework to empirically discerning the presence of consciousness in human patients suffering from unresponsive wakefulness syndrome (UWS)^18^. Subsequently, we provide an example regarding Alzheimer’s disease and the persistence of the self, which illustrates the implications of theoretical frameworks in other areas of research that are related to consciousness studies.

To be clear, we are not here advocating for any particular theory or model that we discuss in these examples. Our only aim is to illustrate the epistemic role theoretical frameworks can play in consciousness research, and how they can be applied to the limited NCC data in order to make further important inferences regarding consciousness. Once this is accepted, the necessary work that will remain is rationally adjudicating between theories and discerning which theory(s) is most likely true and therefore may be employed in the ways suggested. This will inevitably be a long-term task, which will become more viable as research technology and our understanding of the brain progress.

### Unresponsive Patients

There are cases in which patients with UWS come out of an unresponsive state and recall being conscious while previously completely unresponsive. Consider a situation in which a patient gives no behavioral indication of responding to external stimuli. Furthermore, suppose the patient gives no indication *via* brain activity (e.g., neural activity in the motor cortex when the patient is asked to imagine playing tennis). It is possible that the patient, while unresponsive, is nevertheless conscious.

Since medical practitioners do not have access to the patients first person perspective and the patient does not provide any way of verifying her consciousness from a third person perspective *via* a response, an empirical method of verification is needed. The neuronal footprint of consciousness in the form of the full NCC (i.e., the neural correlate of being conscious vs. not being conscious) becomes vitally applicable at this point. However, verifying an NCC in a healthy human subject depends on verbal reports or a corresponding physiological indicator that has been previously verified by a report from the conscious subject (see Tsuchiya et al., 2015); and if it is true that NCC are physically sufficient and zombies are metaphysically or logically possible, how can we know that a full NCC is indicative of consciousness when a patient has undergone brain damage and there is no way for the subject to give us any verification?

A theoretical framework might provide an answer, assuming that the framework is itself warranted. Suppose that there is neural activity in the brain of our unresponsive patient in the posterior cerebral cortex, amongst a coalition of neurons in a temporo-parietal-occipital area^19^. Suppose further that the neural activity is localized, there are no long-range action potentials sent from this coalition of neurons to other brain regions. Assume, however, that the activity is reciprocal and intrinsic within the coalition of neurons. To demonstrate the epistemic utility of a theoretical framework in such cases, let us contrast two theories—the GNW theory and the Integrated Information Theory—and what they would imply in this case based on the above stipulated neurobiological data.

The GNW theory of consciousness is a computational theory (see Dehaene et al., 2017, p. 492)^20^. The theory’s originator, Bernard Baars, hypothesized that within the human brain there is a global workspace that houses information and makes it available to the specialized processing systems throughout the brain (see Baars, 1988). Since the workspace’s capacity is limited, various information signals compete for the privileged position of being the globally available representation in the workspace. That which comes to occupy the workspace is conscious; the signals that do not make it into the workspace are not conscious. Contemporary proponents of GNW have applied the theory to the neurophysiology of the neocortex (see Dehaene and Changeux, 2011; Dehaene, 2014). Per GNW, an indicator of consciousness is a global broadcast of information involving the activity of a prefrontal-parietal network of long-range cortical neurons corresponding with activity in high-level sensory cortices that receive the broadcast. This makes the information globally available for various functional processes (e.g., speech, memory, action) and thus conscious content, according to GNW.

Given the neurobiological data stipulated above regarding the unresponsive patient, a prognosis guided by the GNW theory would declare the patient unconscious. After all, the localized neural activity makes no long-range projections. Nothing about the description of the scenario suggests there is information being processed that is globally available to areas throughout the brain for corresponding function, which is required for consciousness, according to GNW. On the other, a prognosis based on the same data that is instead guided by the Integrated Information Theory of consciousness (for brevity IIT) might be more optimistic in this case.

IIT was developed by the Italian psychiatrist and neuroscientist, Giulio Tononi. It starts with five self-evident axioms about the nature of consciousness and infers corresponding postulates about the nature of the physical substrate of consciousness. In short, according to this theory, consciousness involves information that is integrated, and the physical substrate of consciousness is also integrated in that it exemplifies a structure in the central nervous system that exhibits a maximal intrinsic cause-effect power called *Phi* and symbolized by φ. This power manifested by the physical substrate consisting of a causal structure in the central nervous system is consciousness. Thus, given a causal structure that manifests an intrinsic causal power in the central nervous system, consciousness is present because it is the causal power being manifested, according to IIT. Some leading proponents of IIT aim to develop a consciousness meter capable of measuring a patient’s level of consciousness by measuring the intrinsic causation manifested in the cortex (see Massimini and Tononi, 2018; Koch, 2019). The greater the φ measurement, the higher the level of consciousness. Likewise, a lower φ measurement indicates a lower level consciousness, and a negative measurement indicates unconsciousness. Yet as long as there is a positive φ measurement, which indicates intrinsic causation manifested in the cortex, consciousness is present.

Given the neurobiological data stipulated above regarding the unresponsive patient, a prognosis guided by IIT would be based on a measurement of the intrinsic causation manifested by the localized reciprocal neural activity. The reciprocal nature of the neural activity would be worth investigating to see if there is a manifestation of intrinsic causation present. If it turned out that there was some degree, however minor, of positive φ then consciousness is present, according to IIT; whereas according to GNW this would not be sufficient for consciousness.

This example illustrates the practical difference two different theoretical frameworks can make to a medical prognosis regarding consciousness in unresponsive human patients^21^. By extension, one could see how the theories might be extrapolated to empirically test for consciousness in other biological organisms with a nervous system similar to ours. The GNW theorist would look for a global workspace with information available to other brain regions for corresponding function, whereas the IIT theorist would look for intrinsic causation. There is also interest in applying these theories to evaluating alleged machine consciousness (see Dehaene et al., 2017; Koch and Tononi, 2017). Certainly, applying such theories beyond biological nervous systems is more speculative at this point and caution is in order (see Owen, 2019a). Moreover, there is more immediate applicability pertaining to human consciousness beyond just discerning the presence of consciousness. There are also implications for neighboring fields of study. For an example relevant to psychology, let us consider theoretically informed ways of thinking about Alzheimer’s disease and the persistence of the self.

### Alzheimer’s and the Self

When medial temporal lobe structures vital to the formation and retention of long-term memories become dysfunctional because of plaque, tangles, and other molecular detritus of advanced dementia, autobiographical episodic memories of Alzheimer’s patients are often irretrievably lost^22^. Does this loss of memory about one’s past experiences also entail a loss of the self, according to which the person who once was discontinues? Different theoretical frameworks motivate different answers.

One answer is that the self discontinues when the neural correlates supporting conscious recall of autobiographical episodic memories no longer function properly and access to such memories is lost. This conclusion is motivated by a psychological continuity view of personal identity often attributed to John Locke and his widely influential work *An Essay Concerning Human Understanding* [see especially (Locke, 1975) Bk II, Ch. 27, sect. 19–20]. A person’s ontological identity is thought to be grounded in the individual’s psychological continuity of past experiences that requires conscious recall of autobiographical episodic memories. Therefore the loss of conscious access to such memories that is the result of dysfunctional neural correlates underpinning that access entails the loss of the self, or the person, that once was. The empirical data garnered from brain imaging about the neural correlates does not by itself justify such a conclusion, far from it. Rather, such a conclusion is inescapably theory loaded, based on the idea that psychological continuity is what grounds the persistence of the person.

However, the conclusion that the self is lost does not follow given a different theoretical framework such as the Mind-Body Powers model of NCC (Owen, 2018a). According to the human ontology informing the model, a human person is a substance with mental powers, such as the capacity to consciously recall autobiographical episodic memories, that requires the co-manifestation of particular bodily powers. The bodily powers that are the partner-powers of mental powers require adequate biological structures at the neuronal level that manifest the requisite bodily powers. The loss of or damage to neural tissue that manifest such bodily powers that must be co-manifested with particular mental powers for their full exercise can imply that the person loses their ability to naturally exercise the mental power. But this change in the substance—i.e., the human person—actually requires that the substance persist through the change, otherwise the substance would not actually be undergoing change but just cease to exist. This framework allows for the continuity of the person. The individual continues to exist, but takes on the attribute of being mentally impaired in that the individual cannot manifest a mental power she was once capable of manifesting (i.e., the conscious recall of autobiographical memories). A person who becomes paraplegic due to damage to their spinal cord persists despite losing a bodily power that required the neural architecture lost. Likewise, given the framework of the Mind-Body Powers model of NCC, an Alzheimer’s patient can continue to exist despite a loss of the neural substrate needed to exercise a mental power.

It may turn out to be a case that the theoretical frameworks discussed in this section are false or require significant development and alterations. We are not here advocating for any particular theory. The utility of a theoretical approach in consciousness research and related fields is our concern. Given that NCC are physically sufficient for corresponding consciousness, there are limits to just how much the search for NCC will tell us about the nature of consciousness, what is conscious, and what consciousness indicates about human ontology. Therefore, a reliable theoretical framework can provide additional premises upon which we can reach further conclusions given the data verified *via* NCC research. Clearly, a crucial step at this point in consciousness research is discerning which theories are viable and therefore can be reliably applied to interpreting NCC data. This will require extensive research testing various theoretical frameworks on multiple grounds.

## Philosophically Polluting Consciousness Science?

Before concluding, let us address a concern that those who think consciousness research must always begin with the empirical data might have about the suggested theoretical approach. The concern is that, whether the advocates of theories acknowledge it or not, theoretical frameworks include philosophical tenets that would be part of a theoretical starting point, and this could compromise the empirical rigor of the science of consciousness. For example, GNW appeals to a computational or functionalist view of consciousness that is a view in philosophy of mind (see Dehaene et al., 2017, p. 492). Moreover, IIT’s starting axioms are allegedly directly known from the first-person subjective perspective and they are not known from a third-person perspective observing the brain (see Oizumi et al., 2014; Tononi et al., 2016). And the psychological continuity view of the self pertains to human ontology, while the Mind-Body Powers model appeals to an Aristotelian powers ontology (see Owen, 2018a, section 2).

We acknowledge that philosophical, pre-empirical ideas play an important role in this approach. However, the idea that consciousness research must begin with the raw empirical data unadulterated by philosophical principles is itself a pre-empirical, philosophical idea and it is difficult to see how it could be empirically validated. Thus, consciousness research founded on this ideological foundation is foundationally informed by an epistemological principle about consciousness research methodology. It is guilty of the very charge its advocate levels against the theoretical approach to consciousness research. Yet, what matters is not whether philosophical tenets inform our consciousness research, but whether the tenets we allow to inform our research are rationally valid (see Guta, 2015). And if we do not acknowledge the role such tenets play in a research approach then we are more likely to be guided by unacknowledged, unanalyzed, and untested philosophical principles that can influence our interpretations of empirical data. By contrast, the theoretical approach makes explicit the ideological starting points that are informing our research methods, data acquisition, and interpretations. This not only makes the influence of such starting points more apparent but also makes it easier to scrutinize them and to evaluate the legitimacy of their influence. So those worried about philosophical tenets watering down empirical rigor in consciousness research would actually benefit from adopting the theoretical approach we are advocating.

On our view, proposed theoretical frameworks should be evaluated on multiple grounds. For one, the logical coherence of the framework must be rigorously analyzed. It is fiscally prudent to do this at the beginning of the evaluation process, since logical analysis of a theory’s internal coherence is often less costly than empirical research requiring expensive technology. A thorough logical analysis of a theory might expose a fatal logical inconsistency, allowing researchers and funding bodies to avoid spending valuable resources on empirically testing an incoherent theory that’s not possibly true (see Tahko, 2012, pp. 39–42). However, multiple competing theories can be internally coherent. Therefore, theoretical virtues used to evaluate the legitimacy of scientific theories in general should be used to evaluate competing theoretical frameworks in consciousness research. To use the example theories discussed above in sections Must the correlation hold across all cases or only across specific types of cases (ie., cases with ordinary brain function in an ordinary environment, cases with a normal brain but unusual inputs, cases with varying stimulation, or cases with abnormal brain function due to lesions)?, researchers should consider whether GNW or IIT has more theoretical virtues. Does one theory have more explanatory scope or explanatory power? Is IIT or GNW simpler, in that one is more capable of explaining relevant data without postulating unnecessary entities? Which theory coheres best with widely accepted theories in related research fields? Such questions can be used to weed out inadequate theories with less theoretical virtues and to identify more promising, theoretically virtuous, theories.

After analyzing competing theories based on logical coherence and theoretical virtues, multiple theories might remain viable contenders, but empirical research can be used to confirm or falsify remaining candidates. The empirically testable predictions that are implied by a theory must be tested, with the outcomes confirming or disconfirming the theory. Although this immensely valuable work will be empirical, here too, logical and philosophical analysis is useful. It can clarify which predictions are strictly entailed by the theory, probably in light of the theory, merely consistent with the theory, or simply an opinion of an advocate of the theory that’s not part of the theory itself. Furthermore, philosophical analysis can clarify what aspects of consciousness are relevant to particular predictions and what the implications are. For example, philosophers often distinguish between access and phenomenal consciousness, which is relevant to the hypothetical case above involving an unresponsive patient and the applicability of IIT and GNW^23^. For GNW, one might think, concerns access consciousness whereas IIT concerns phenomenal consciousness and therefore this must be remembered when employing or comparing the theories and their predictions. One might even object to the use of IIT and GNW in our example on the grounds that the two theories are not about “consciousness” in the same sense. This is a fair point, which actually highlights the importance of philosophical analysis to a theoretical approach.

While this approach can potentially advance consciousness research, we make no claim that it will make it easier. In addition to arduous empirical testing, responsible theoretically motived consciousness research requires rigorous philosophical analysis to ensure that logically coherent, theoretically virtuous, and empirically adequate theories are recognized as such, and the opposite are discarded.

## Results

After analyzing three relevant senses of “sufficient” it appears that the definition of NCC is best understood according to physical sufficiency. Yet if NCC are physically sufficient for consciousness, there are limits to what NCC data alone can tell us in certain areas of consciousness research. A way of overcoming these limitations is to take a theoretically-motivated approach to certain issues. Once proven viable, a theory can provide additional information that can provide grounds for making further inferences in light of data about NCC. In the long-term, properly understanding NCC will strengthen the vitality of the search for NCC, enabling it to empirically progress unhindered by philosophical debates about the nature of consciousness. While there are limits to what this search can do, one thing it has done and will continue to do is foster scientific interest in consciousness studies.

## Data Availability

All datasets analyzed for this study are included in the manuscript and the supplementary files.

## Author Contributions

MO formulated the argument and wrote the manuscript incorporating feedback provided by MG in substantial comments on a prior version.

## Conflict of Interest Statement

The authors declare that the research was conducted in the absence of any commercial or financial relationships that could be construed as a potential conflict of interest.

## Footnotes

^1^See also Chalmers (2010, ch. 3).

^2^Fink’s claim that “none of the mentioned authors quotes Chalmers (2000)” seems mistaken, as Mormann and Koch (2007) and Bayne and Hohwy (2013, p. 25) cite Chalmers (2000), as does Block (2005, p. 47) although it’s misdated in Block’s bibliography.

^3^There is debate about whether this is Hume’s view (see Beebee, 2006). Here, we are simply using a common conception of his view as an example to illustrate a point.

^4^Metzinger (2000a, pp. 4-5) discusses relevant neutrality.

^5^Personal correspondence 26 December 2016.

^6^Phenomenal consciousness pertains to the felt sensation of what it is like to be in a particular conscious state, whereas access consciousness pertains to the accessibility of a conscious state for active use in reasoning, recall, speech, and the like (see Block, 1995, pp. 230–232). Phenomenal consciousness can be (and some would say always is) access consciousness as well.

^7^See Muñoz-Cespedes et al. (2005), Jensen and Overgaard (2011), Mogensen, 2011, Overgaard and Mogensen (2011).

^8^Owen is indebted to Christof Koch for this point.

^9^This general definition can be modified to fit specific classes of phenomenal consciousness (see Chalmers, 2000, p. 31).

^10^For further clarification, see Owen (2019b, pp. 242–245).

^11^The qualifier regarding clones is an extrapolation from what is known about cloned mice.

^12^Owen is indebted to Koch here too.

^13^For various reservations about this inquiry, see Hardcastle and Stewart (2009), Shulman (2013) and Tsuchiya et al. (2015).

^14^See also Koch (2004, ch. 12), and Koch (2012, ch. 6).

^15^By ‘law’ we merely mean consistent regularities throughout the physical world. There are various ways of ontologically describing and accounting for such regularities (see Hume, 2007, section VII; Heil, 2012, ch. 6).

^16^See Crick and Koch (1990).

^17^Hence Frith and Rees (2017 p. 3) can claim Descartes “…was the first to think seriously about the neural correlates of consciousness” (see Koch, 2015).

^18^Also called a vegetative state (see Laureys et al., 2010).

^19^Koch et al. (2016a,b) discuss a posterior cortical “hot zone” in this area.

^20^For a lucid summary of the theory which has informed our own synopsis here, as well as an objection to it, see Koch (2019, Ch. 13).

^21^Here there are multiple related ethical questions that require carefull consideration, which we are indebted to a reviewer for raising. For one, how much justification must a theory have before we allow it to inform medical decisions? When theories do play a role, how much preference, or weight, should be given to them in the decision-making process? When there are different potential diagnoses informed by different theories of consciousness, who should have the final say about whether a patient is or is not conscious? These are very important questions that we want to note here but will leave to be addressed by forthcoming works.

^22^Owen is indebted to Koch here, and we also benefited from insightful comments from a referee for this journal.

^23^On access versus phenomenal consciousness, see footnote 4 above.

## References

[B1] Aru J. Bachmann T. Singer W. Melloni L. (2012). Distilling the neural correlates of consciousness. Neurosci. Biobehav. Rev. 36, 737–746. 10.1016/j.neubiorev.2011.12.003 22192881

[B2] Baars B. J. (1988). A Cognitive Theory of Consciousness. New York, NY: Cambridge University Press.

[B3] Bailey A. M. Rasmussen J. Horn L. V. (2011). No pairing problem. Philos. Stud. 154, 349–360. 10.1007/s11098-010-9555-7

[B4] Bayne T. Hohwy J. (2013). “Consciousness: theoretical approaches,” in Neuroimaging of Consciousness, eds Cavanna A. E. Nani A. Blumenfeld H. Laureys S. (Heidelberg: Springer), 23–35.

[B5] Beebee H. (2006). Hume on Causation. New York, NY: Routledge.

[B6] Block N. (1995). On a confusion about a function of consciousness. Behav. Brain Sci. 18, 227–247. 10.1017/S0140525X00038188

[B7] Block N. (2005). Two neural correlates of consciousness. Trends Cogn. Sci. 9, 46–52. 10.1016/j.tics.2004.12.006 15668096

[B8] Block N. Stalnaker R. (1999). Analysis, dualism, and the explanatory gap. Philos. Rev. 108, 1–46. 10.2307/2998259

[B9] Boly M. Massimini M. Tsuchiya N. Postle B. R. Koch C. Tononi G. (2017). Are the neural correlates of consciousness in the front or in the back of the cerebral cortex? Clinical and neuroimaging evidence. J. Neurosci. 37, 9603–9613. 10.1523/JNEUROSCI.3218-16.2017 28978697 PMC5628406

[B10] Cartwright N. Pemberton J. (2013). “Aristotelian powers: without them, what would modern science do?” in Powers and Capacities in Philosophy: The New Aristotelianism, eds Groff R. Greco J. (New York, NY: Routledge), 93–112.

[B11] Chalmers D. J. (1995). Facing up to the problem of consciousness. J. Conscious. Stud. 2, 200–219.

[B12] Chalmers D. J. (1996). The Conscious Mind: In Search of a Fundamental Theory. New York, NY: Oxford University Press.

[B13] Chalmers D. J. (1998). “On the search for the neural correlate of consciousness” in Toward a Science of Consciousness II: The Second Tucson Discussions and Debates, eds Hameroff S. R. Kaszniak A. W. Scott A. C. (Cambridge, MA: MIT Press), 219–229.

[B14] Chalmers D. J. (2000). “What is a neural correlate of consciousness?,” in Neural Correlates of Consciousness, ed. Metzinger T. (Cambridge, MA: MIT Press), 17–40.

[B15] Chalmers D. J. (2010). The Character of Consciousness. New York, NY: Oxford University Press.

[B16] Crick F. Koch C. (1990). Toward a neurobiological theory of consciousness. Semin. Neurosci. 2, 263–275.

[B17] Crick F. Koch C. (2003). A framework for consciousness. Nat. Neurosci. 6, 119–126. 10.1038/nn0203-119 12555104

[B18] Dehaene S. (2014). Consciousness and the Brain: Deciphering How the Brain Codes Our Thoughts. New York, NY: Penguin Books.

[B19] Dehaene S. Changeux J.-P. (2011). Experimental and theoretical approaches to conscious processing. Neuron 70, 200–227. 10.1016/j.neuron.2011.03.018 21521609

[B20] Dehaene S. Lau H. Kouider S. (2017). What is consciousness, and could machines have it? Science 358, 486–492. 10.1126/science.aan8871 29074769

[B21] Farahany N. A. Greely H. T. Hyman S. Koch C. Grady C. Pasca S. P.. (2018). The ethics of experimenting with human brain tissue. Nature 556, 429–432. 10.1038/d41586-018-04813-x 29691509 PMC6010307

[B22] Fink S. B. (2016). A deeper look at the “neural correlate of consciousness”. Front. Psychol. 7:1044. 10.3389/fpsyg.2016.01044 27507950 PMC4960249

[B23] Frith C. D. Rees G. (2017). “A brief history of the scientific approach to the study of consciousness,” in The Blackwell Companion to Consciousness, eds Schneider S. Velmans M. (Oxford, UK: Wiley Blackwell), 3–16.

[B25] Guta M. P. (2015). Consciousness, first-person perspective and neuroimaging. J. Conscious. Stud. 22, 218–245.

[B26] Guta M. P. (2019). “Introduction,” in Consciousness and the Ontology of Properties, ed. Guta M. P. (New York, NY: Routledge), 1–12.

[B27] Haldane J. (1998). A return to form in the philosophy of mind. Ratio 11, 253–277. 10.1111/1467-9329.00070

[B28] Hardcastle V. G. Stewart C. M. (2009). “fMRI: a modern cerebrascope? The case of pain,” in The Oxford Handbook of Philosophy and Neuroscience, ed. Bickle J. (New York, NY: Oxford University Press), 179–199.

[B29] Heil J. (2012). The Universe As We Find It. New York, NY: Oxford University Press.

[B31] Hohwy J. (2007). The search for the neural correlates of consciousness. Philos. Compass 2, 461–474. 10.1111/j.1747-9991.2007.00086.x

[B32] Hohwy J. (2009). The neural correlates of consciousness: new experimental approaches needed? Conscious. Cogn. 18, 428–438. 10.1016/j.concog.2009.02.006 19345590

[B33] Hohwy J. Bayne T. (2015). “The neural correlates of consciousness: causes, confounds and constituents,” in The Constitution of Phenomenal Consciousness: Toward a Science and Theory, ed. Miller S. M. (Philadelphia, PA: John Benjamins Publishing Company), 155–176.

[B30] Hohwy J. Frith C. D. (2004). The neural correlates of consciousness: room for improvement, but on the right track: comment. J. Conscious. Stud. 11, 45–51.

[B34] Hume D. (2007). An Enquiry Concerning Human Understanding. ed. Millican P. (New York, NY: Oxford University Press).

[B35] Jensen M. Overgaard M. (2011). Neural plasticity and consciousness. Front. Psychol. 2:191. 10.3389/fpsyg.2011.00191 21909329 PMC3164108

[B36] Kandel E. R. Hudspeth A. J. (2013). “The brain and behavior,” in Principles of Neural Science, 5th Edn. eds Kandel E. R. Schwartz J. R. Jessell T. M. Siegelbaum S. A. Hudspeth A. J. (New York, NY: McGraw-Hill), 5–20.

[B37] Kim J. (2005). Physicalism, Or Something Near Enough. Princeton, New Jersey, NJ: Princeton University Press.

[B38] Koch C. (2004). The Quest for Consciousness: A Neurobiological Approach. Englewood, Colorado: Roberts and Company Publishers.

[B39] Koch C. (2012). Consciousness: Confessions of a Romantic Reductionist. Cambridge, MA: The MIT Press.

[B40] Koch C. (2015). Two natural philosophers, centuries apart, converse about the mind. Sci. Am. Mind 26, 28–31. 10.1038/scientificamericanmind0315-28

[B41] Koch C. (2019). The Feeling of Life Itself. Cambridge, MA: MIT Press.

[B42] Koch C. Crick F. (2001). The zombie within. Nature 411:893. 10.1038/35082161 11418835

[B44] Koch C. Massimini M. Boly M. Tononi G. (2016a). Neural correlates of consciousness: progress and problems. Nat. Rev. Neurosci. 17, 307–321. 10.1038/nrn.2016.22 27094080

[B45] Koch C. Massimini M. Boly M. Tononi G. (2016b). Posterior and anterior cortex—where is the difference that makes the difference? Nat. Rev. Neurosci. 17:666. 10.1038/nrn.2016.105 27466141

[B43] Koch C. Tononi G. (2017). Can we quantify machine consciousness? IEEE SPECTRUM Available online at: http://spectrum.ieee.org/computing/hardware/can-we-quantify-machine-consciousness. Accessed November 29, 2018.

[B46] Koons R. C. Bealer G. (Eds.) (2010). The Waning of Materialism. New York, NY: Oxford University Press.

[B48] Laureys S. Celesia G. G. Cohadon F. Lavrijsen J. León-Carrión J. Sannita W. G.. (2010). Unresponsive wakefulness syndrome: a new name for the vegetative state or apallic syndrome. Eur. J. Neurol. 8:68. 10.1186/1741-7015-8-68 21040571 PMC2987895

[B49] Locke J. (1975). An Essay Concerning Human Understanding. Edited by P. Nidditch. Oxford: Oxford University Press.

[B50] Massimini M. Tononi G. (2018). Sizing Up Consciousness: Towards an Objective Measure of the Capacity for Experience. New York, NY: Oxford University Press.

[B51] Metzinger T. (2000a). “Introduction,” in Neural Correlates of Consciousness: Empirical and Conceptual Questions, ed. Metzinger T. (Cambridge, MA: The MIT Press), 1–12.

[B52] Metzinger T. (Ed.). (2000b). Neural Correlates of Consciousness: Empirical and Conceptual Questions. Cambridge, MA: The MIT Press.

[B53] Mogensen J. (2011). Reorganization of the injured brain: implications for studies of the neural substrate of cognition. Front. Psychol. 2:7. 10.3389/fpsyg.2011.00007 21713186 PMC3111425

[B54] Mormann F. Koch C. (2007). Neural correlates of consciousness. Scholarpedia 2:1740 10.4249/scholarpedia.1740

[B55] Muñoz-Cespedes J. M. Rios-Lago M. Paul N. Maestu F. (2005). Functional neuroimaging studies of cognitive recovery after acquired brain damage in adults. Neuropsychol. Rev. 15, 169–183. 10.1007/s11065-005-9178-5 16395622

[B56] Oizumi M. Albantakis L. Tononi G. (2014). From the phenomenology to the mechanisms of consciousness: integrated information theory 3.0. PLoS Comput. Biol. 10:e1003588. 10.1371/journal.pcbi.1003588 24811198 PMC4014402

[B57] Overgaard M. (2017). The status and future of consciousness research. Front. Psychol. 8:1719. 10.3389/fpsyg.2017.01719 29066988 PMC5641373

[B58] Overgaard M. Mogensen J. (2011). A framework for the study of multiple realizations: the importance of levels of analysis. Front. Physiol. 2:79. 10.3389/fpsyg.2011.00079 21772823 PMC3131792

[B59] Owen M. (2018a). Aristotelian causation and neural correlates of consciousness. Topoi 1–12. 10.1007/s11245-018-9606-9

[B62] Owen D. (2018b). “Neo-Thomistic hylomorphism applied to mental causation and neural correlates of consciousness,” in Doctoral dissertation (Birmingham, UK: University of Birmingham).

[B60] Owen M. (2019a). Exploring common ground between integrated information theory and aristotelian metaphysics. J. Conscious. Stud. 26, 163–187.

[B61] Owen M. (2019b). “Neural correlates of consciousness and the nature of the mind,” in Consciousness and the Ontology of Properties, ed. Guta M. P. (New York, NY: Routledge), 241–260.

[B63] Penfield W. (1938). The cerebral cortex in man: the cerebral cortex and consciousness. Arch. Neurol. Psychiatry 40, 417–422. 10.1001/archneurpsyc.1938.02270090011001

[B64] Penfield W. (1975). The Mystery of the Mind: A Critical Study of Consciousness and the Human Brain. Princeton, NJ: Princeton University Press.

[B65] Shulman R. G. (2013). Brain Imaging: What It Can (and Cannot) Tell Us About Consciousness. New York, NY: Oxford University Press.

[B66] Storm J. F. Boly M. Casali A. G. Massimini M. Olcese U. Pennartz C. M. A.. (2017). Consciousness regained: disentangling mechanisms, brain systems, and behavioral responses. J. Neurosci. 37, 10882–10893. 10.1523/jneurosci.1838-17.2017 29118218 PMC5678021

[B67] Swinburne R. (2013). Mind, Brain and Free Will. Oxford: Oxford University Press.

[B68] Tahko T. E. (2012). “In defence of aristotelian metaphysics,” in Contemporary Aristotelian Metaphysics, ed. Tahko T. E. (New York, NY: Oxford University Press), 26–43.

[B70] Tononi G. Boly M. Massimini M. Koch C. (2016). Integrated information theory: from consciousness to its physical substrate. Nat. Rev. Neurosci. 17, 450–461. 10.1038/nrn.2016.44 27225071

[B69] Tononi G. Koch C. (2008). The neural correlates of consciousness: an update. Ann. N Y Acad. Sci. 1124, 239–261. 10.1196/annals.1440.004 18400934

[B71] Tsuchiya N. Wilke M. Frässle S. Lamme V. A. F. (2015). No-report paradigms: extracting the true neural correlates of consciousness. Trends Cogn. Sci. 19, 757–770. 10.1016/j.tics.2015.10.002 26585549

[B24] van Gulick R. (2017). “Consciousness,” in Stanford Encyclopedia of Philosophy (Summer 2017 Edition), ed. Zalta. E. N. Available online at: https://plato.stanford.edu/archives/spr2018/entries/consciousness/

[B72] Willyard C. (2015). Rise of the organoids: biologists are building banks of mini-organs and learning a lot about human development on the way. Nature 520–522.

